# Clinico-radiological outcomes of desmoid type-fibromatosis after discontinuing the sorafenib treatment in responders – early results from the SORASTOP study

**DOI:** 10.3332/ecancer.2025.1915

**Published:** 2025-05-27

**Authors:** Bharath B Gangadharaiah, Ghazal Tansir, Sameer Rastogi, Simran Kaur, Vikas Garg, Ekta Dhamija, Adarsh Barwad, Shivanand Gamanagatti, Sandeep Bhoriwal, Maroof A Khan

**Affiliations:** 1 Department of Medical Oncology, Institute Rotary Cancer Hospital, All India Institute of Medical Sciences, New Delhi 110029, India; 2 Department of Physiology, All India Institute of Medical Sciences, New Delhi 110029, India; 3 Department of Radio-diagnosis, All India Institute of Medical Sciences, New Delhi 110029, India; 4 Department of Pathology, All India Institute of Medical Sciences, New Delhi 110029, India; 5 Department of Surgical Oncology, Institute Rotary Cancer Hospital, All India Institute of Medical Sciences, New Delhi 110029, India; 6 Department of Biostatistics, All India Institute of Medical Sciences, New Delhi 110029, India

**Keywords:** desmoid-type fibromatosis, sorafenib, tyrosine kinase inhibitors, quality of life, cognitive function

## Abstract

**Introduction:**

The duration of treatment for desmoid-type fibromatosis (DTF) is undefined. This study aimed to evaluate the efficacy of discontinuing sorafenib in responding patients with extremity DTF. We hereby report the initial findings comprising outcomes of 20 evaluable patients enrolled in this study.

**Methods:**

This prospective single-arm phase 2 Simon’s 2-stage trial enrolled adults with radiologically non-progressive, pain-free (Edmonton Symptom Assessment Scale (ESAS) score <2) extremity DTF post at least 1 year of sorafenib. Sorafenib was discontinued and patients were monitored by clinical examination, magnetic resonance imaging, European Organisation for Research and Treatment of Cancer Quality of Life Questionnaire-Core 30 and Addenbrooke’s Cognitive Examination. Disease progression was defined as ≥10% increase in size plus ESAS >5 or ≥20% increase in size. The primary endpoint was a 1-year progression-free survival rate (PFR) after discontinuation. An unplanned analysis of the primary objective among 20 evaluable patients is being presented in this study.

**Results:**

33 patients had a median age of 29.5 years (range: 23–38) and a female-to-male ratio of 1.2:1. Median duration of sorafenib therapy was 24 months (range: 14.5–33.5), and at a median follow-up of 15 months (range: 9–18), 20 patients were evaluable. Among the 20 evaluable patients, 1-year change in tumour size ranged from a 21% decrease to a 32% increase. Three patients restarted sorafenib because of pain with stable disease (*n* = 2) and radiological progression (*n* = 1). 6-month and 1-year PFR was 96.7% and 95%, respectively. Statistically significant quality of life (QoL) improvement was demonstrated in insomnia (*p* = 0.01), diarrhea (*p* = 0.02), physical (*p* < 0.001) and social (*p* = 0.04) functioning at 12 months while neurocognitive functions remained stable.

**Conclusion:**

As per the early results, stopping sorafenib can be potentially considered in responding patients with stable extremity DTF after at least 1 year of treatment. With improvement in QoL and an acceptable rate of disease progression upon stopping sorafenib, this treatment discontinuation strategy could be an important consideration in DTF management. Further analysis of the entire study cohort is warranted to establish optimal treatment duration for extremity DTF.

## Introduction

Desmoid-type fibromatosis (DTF) is a rare benign tumour occurring in young adults, particularly in females [1, 2]. The locally infiltrating disease is associated with morbidity attributable to pain, functional limitations and psychosocial issues [3]. The treatment of DTF poses a significant clinical conundrum, given the varying management guidelines [4]. While active surveillance is recommended for asymptomatic cases owing to spontaneous regression reported in up to 20% of patients, surgical resection is avoided given the high rates of post-surgical recurrences [4]. Tyrosine kinase inhibitors (TKIs) and the recently approved γ-secretase inhibitor (nirogacestat) comprise the current medical therapies used in the management of DTF [5, 6].

Treatment with sorafenib is associated with adverse effects such as fatigue, hand-foot skin reaction, hypertension, gastrointestinal disturbances and deterioration in quality of life (QoL) [5]. Data regarding its long-term toxicities is derived from other cancers with limited life expectancy, thus late effects involving cognition, cardiovascular and reproductive functions are unknown. A study among patients with gastrointestinal stromal tumour and renal cell carcinoma, found greater effects of vascular endothelial growth factor (VEGF) inhibitors on learning, memory and executive functions as compared to controls [7]. VEGF inhibitors are also associated with hyperlipidemia and cardiac problems that might harm the longevity of these young patients [8].

The chronic, slow-growing behaviour of DTF combined with the side effects of long-term TKI use raises the question of discontinuation of therapy. Only the DESMOPAZ study among patients with DTF exhibited the efficacy of fixed-duration pazopanib prescribed for 1 year, producing a 6-month progression-free survival rate (PFR) of 83.7% [9].

We conducted the first prospective study assessing the progression-free response, cognitive profile and QoL after sorafenib discontinuation in patients with extremity DTF who achieved disease stabilisation following at least 1 year of therapy. We have obtained promising results from the initial analysis and hereby present these findings which can aid in optimising the treatment duration of stable extremity DTF.

## Methods

### Study population

The study included consenting patients over 18 years of age with histologically confirmed extremity DTF, who had received sorafenib for at least 1 year and were symptom-free at the time of recruitment. Symptom-free status was defined in the study protocol as the Edmonton Symptom Assessment Scale (ESAS) score of less than 2 for pain [10].

### Trial oversight

The trial was conducted at the Department of Medical Oncology at All India Institute of Medical Sciences, New Delhi, between July 2021 and June 2023. The study was approved by the Institute’s ethics committee (IECPG-430/28.07.2021) and all participants provided informed consent.

### Trial design and treatment

This was a prospective single-arm phase 2 clinical trial following Simon’s two-stage phase 2 design. Eligible patients underwent baseline clinical assessment, including medical history and physical examination before sorafenib was discontinued for the study.

The following assessments were performed during the study (Table 1):

1. Clinical assessment by 3 month history and physical examination.
2. Radiological assessment by magnetic resonance imaging (MRI) scans of the tumour area were conducted 6 months to assess tumour size and response to treatment or immediately if patients reported recurrence in pain with an ESAS score of ≥5. Change in tumour dimension was calculated using Response Evaluation Criteria in Solid Tumours version 1.1 (RECIST v1.1) [11].
3. QoL: The European Organisation for Research and Treatment of Cancer Quality of Life Questionnaire-Core 30 (EORTC QLQ-C30) was used with scoring by the EORTC manual [12]. This has been previously utilised for QoL assessment in patients with DTF in a study published by our center [13]. The questionnaire was administered in Hindi or English as per the participants’ comprehension at 3 month intervals with scoring between one and four. It included a global health status scale, five functional scales (physical, role, cognitive, emotional and social functioning), three symptom scales (fatigue, nausea, vomiting and pain) and six single items (appetite loss, diarrhea, dyspnea, constipation, insomnia and financial difficulties).
4. Symptom assessment: ESAS pain scale was used to quantify pain intensity and its impact on daily activities. ESAS is a validated tool commonly used to assess pain severity in oncology settings [14].
5. Cognitive assessment: The Addenbrooke’s Cognitive Examination III (ACE-III) questionnaire was used, which is a validated tool for assessing cognitive impairment. It has been widely used in clinical practice and research settings, including in patients with sarcomas at our institution [15, 16]. The amendment to the secondary objective was made after 1 year of starting the study to include the assessment of neurocognitive functions in the cohort.

### Endpoints and assessments

1. Primary objective: Estimation of the proportion of patients experiencing progression upon sorafenib discontinuation after at least 12 months of treatment. Progression was defined by 1-year PFR, with progression criteria including ≥10 percent increase in size along with symptoms (ESAS ≥5 for pain) or ≥20% increase in size irrespective of symptoms by RECIST v1.1.
2. Secondary objectives: 3-month assessment of QoL using the EORTC QLQ C-30 questionnaire and cognitive function using the ACE III questionnaire.

### Sample size and statistical analysis

Sample size calculation was based on an assumed 12-month PFR of 70% upon sorafenib discontinuation compared to historical data with a 12-month PFR of 46% with placebo [5]. Simon’s optimal two-stage, one-sample, testing procedure was applied, with a type 1 (alpha) error of 0.05 and power of 90% [17]. The total sample size required after calculation was 44, rounded off to 50 to account for 10% attrition. The study utilised Simon’s optimal two-stage phase 2 design. As per the study requirement, the study would enter the 2nd stage when at least 8 out of 16 patients would remain progression-free at the end of stage 1. The response rate exceeded this threshold, and the study proceeded to the second stage.

Descriptive statistics provided summaries of the data, including means, standard deviations and proportions, to characterise the QoL and cognitive function scores at various time points after sorafenib discontinuation. Statistical significance was determined using appropriate tests, such as the chi-square test or Fisher’s exact test, depending on the distribution of the data and sample size.

## Results

**Figure 1.**
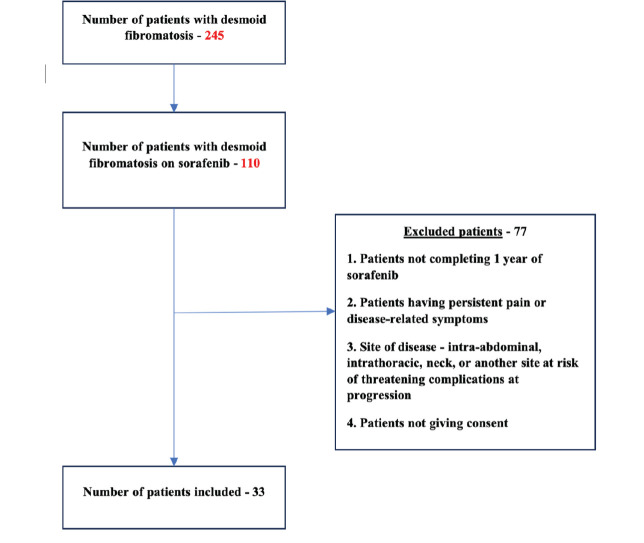
The flow diagram of the SORASTOP study.

245 patients with DTF were screened, among whom 110 patients were on sorafenib. After excluding patients who did not meet the eligibility criteria (Figure 1), 33 patients have been included in the study so far. 20 out of 33 patients have completed the follow-up duration for assessment of the primary objective. The results obtained from this evaluable population are being described here as part of an unplanned interim analysis.

### Patient characteristics

16 patients were enrolled in the first stage of Simon’s two-stage design from July 2021. At the end of the first stage, the study passed the test of futility and continued to enroll 33 patients till the last follow-up date of 30th June 2023. The trial is continuing the enrolment of patients at the time of the first analysis.

The overall study population (*n* = 33) had a median age of 29.5 years (IQR 23–38) with female predominance (*n* = 18, 54.5%) (Figure 1, Table 2). Primary sites of disease included upper limb and shoulder girdle (*n* = 16, 48.4%), lower limb or pelvic girdle (*n* = 13, 39.4%) and superficial trunk (*n* = 4, 12.2%).

At the onset of sorafenib-based therapy (*n* = 33), 13 patients (39.4%) had primary tumours, 9 (27.3%) had tumours recurring after prior surgery and the remaining 11 (33.3%) had refractory disease (progressive disease after one or more medical therapies). Median duration of sorafenib was 24 months (IQR 13.5–33.5) at the most frequently prescribed dose of 200 mg once daily (*n* = 16, 48.5%). At enrollment, 23 patients (69.7%) had stable disease, 9 (27.3%) had a partial response and 1 (3%) had a complete response on sorafenib.

### Outcome measurements

1. Primary outcome

a. Radiological assessment:

Among 33 patients included in the current analysis who had at least one interval follow-up, 20 completed all three MRI assessments (baseline, 6th and 12th month) or had an event, making them eligible for evaluation.

**Figure 2.**
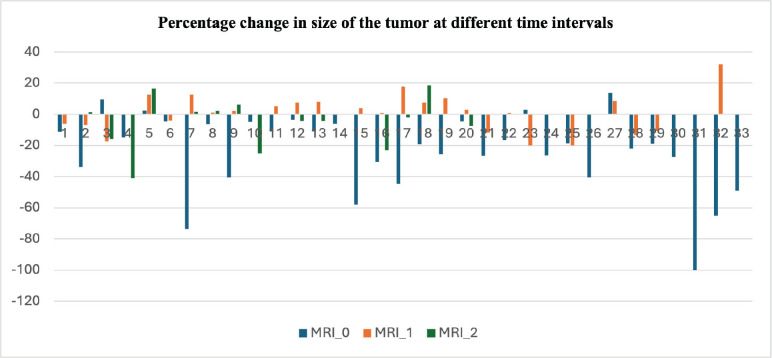
Clustered column chart showing the percentage change in the size of the tumour at three different time points. The blue line (MRI_0) represents the change in the tumour at the time of stopping sorafenib compared to the size at the time of starting sorafenib. The orange line (MRI_1) represents the percentage change in size at 6–9 months after stopping sorafenib compared to the size at the time of inclusion in the study. The grey line (MRI_2) represents the percentage change at 12–15 months compared to the size at the time of inclusion in the study. The negative value is the percentage decrease in the size and the positive value represents the percentage increase in size. The data shown is for all 33 patients evaluated in the study. Abbreviation: Magnetic Resonance Imaging.

**Figure 3.**
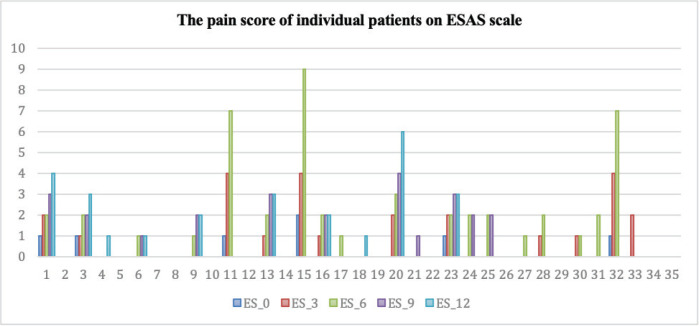
Clustered column chart showing the actual pain scored by the individual patients at 3 different time points on the ESAS. The dark blue line (ES_0) represents the score at the time of stopping sorafenib. The orange line (ES_1) represents the score in 3rd month after stopping sorafenib compared to the size at the time of inclusion in the study. The grey line (ES_2) represents the score in the 6th month. The yellow line (ES_4) is the score in the 9th month and the light blue line (ES_5) is the score in the 12th month. The data shown is for all 33 patients evaluated in the study. Abbreviation: ESAS – Edmonton Symptom Assessment Scale.

Among the 20 patients eligible for primary outcome evaluation at a median follow-up duration of 18 months (IQR 15.25–20.75), 1 (5%) met the RECIST v1.1 criteria for disease progression. 2 (10%) patients withdrew from the study at the 6th and 7th month, respectively, due to recurrent pain and 1 (5%) was lost to follow-up after the 3rd-month assessment. The remaining 16 (80%) who completed the 1-year study duration remained progression-free (Figure 2, Table 2).

b. Symptom assessment for pain:

Among the 20 evaluable patients, 8 (40%) remained symptom-free at the end of 1 year, as indicated by ESAS pain scores (Figure 3). Of the remaining 12 (60%) patients who experienced any increase in the ESAS pain scale, 4 (20%) had a score of ≥5 points.

Among these four patients, one exhibited a tumour dimension increase exceeding 10% and was restarted on sorafenib. Among the three patients who did not meet the criteria for disease progression, two withdrew consent and were restarted on sorafenib, while one patient received supportive management. All three patients who were restarted on sorafenib found complete pain relief.

c. Overall primary outcome analysis

**Figure 4.**
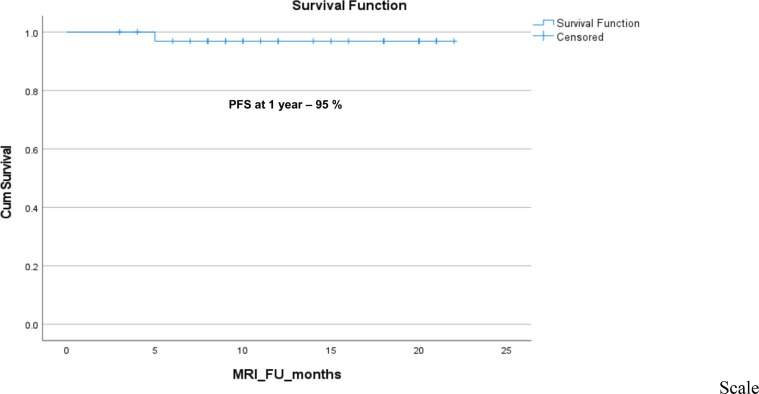
The Kaplan–Meier survival analysis for progression in the desmoid tumour after stopping sorafenib treatment.

Out of 20 evaluable patients, 1 (5%) experienced disease progression after discontinuing sorafenib, as determined by both RECIST v1.1 and ESAS scoring criteria. Among the remaining 19 patients, 3 (15.8%) were censored at the time of restarting sorafenib: two due to recurrence of pain and one due to being lost to follow-up. The 1-year PFR was 95% (Figure 4) and among 29 patients evaluable for 6-month PFR, the 6-month PFR was 96.7%.

**Figure 5.**
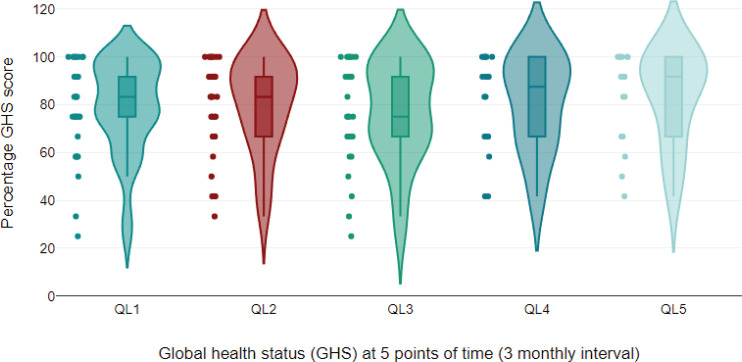
Violin plot of global health status parameter of EORTC QLQc30 scale at different time points; This violin plot illustrates the distribution of percentage GHS (Global Health Score) over five points in time, measured at 3-month intervals. The plot provides a visual summary of the data’s distribution, showing the density and spread of GHS scores at each time point. Violin Shape: Represents the distribution of GHS scores at each time point. Wider sections indicate a higher density of scores at that value. Dot: The GHS score. Thick bar: The interquartile range (IQR), indicates the middle 50% of the data. Thin line: The range of the data, excluding outliers. Coloured regions: Represent the kernel density estimate of the scores, with different colours used to distinguish between time points.

2. Secondary outcomes

a. Health-related QoL:

The EORTC QLQ-C30 questionnaire was filled by 33 patients (100%) at baseline, 31 patients (94%) at 6 months and 17 patients (51.5%) at 12 months (Table 3). At the end of 12 months, there was more than a ten-point improvement in fatigue (*p* = 0.153), insomnia (*p* = 0.011), nausea (*p* = 0.439), diarrhea (*p* = 0.024), anorexia (*p* = 0.083) and financial difficulties (*p* = 0.032) after stopping sorafenib. Among the functioning scales, physical (*p* = <0.001) and social functioning (*p* = 0.049) significantly improved with sorafenib discontinuation. The mean global health status score at the time of stopping sorafenib was 77.24 (Table 4, Figure 5).

b. Neurocognitive examination:

At the time of the current analysis, 16 (48.5%) patients of the total 33 were included in the analysis using the ACE III questionnaire. Out of 16 patients, 6 (37.5%) patients completed a 1-year follow-up and the remaining 10 patients are still on follow-up and have completed the 6th-month assessment. The current analysis showed no statistically significant difference in any parameter of neurocognitive functions at the 6th month or 12th month (Table 5).

## Discussion

This is the first prospective study to address the uncertainty surrounding the optimal duration of sorafenib treatment in extremity DTF. Clinical trials have reported significant treatment discontinuation rates reaching up to 20% for sorafenib and nirogacestat due to adverse events [5, 6]. Our findings indicate that 95% of evaluable patients remained free of disease progression, and 80% did not experience a significant increase in pain 1 year after discontinuation of sorafenib.

Unlike other malignancies, DTF exhibits a notable discordance between radiological progression and symptomatic manifestations. This was demonstrated in a previous study wherein a substantial proportion of patients with DTF exhibited symptomatic progression despite radiological stability or even regression of the tumour [18]. Another retrospective analysis reported that symptomatic relief did not consistently align with radiological response to treatment in patients with DTF undergoing systemic therapy [4]. In our study, about 60% of patients experienced recurrent pain despite the absence of radiological progression. Thus, symptom progression or alleviation in DTF can occur independent of radiological response.

We defined the criteria for disease progression as a 20% increase in tumour size or a 10% change accompanied by an improvement of at least five points in the ESAS score in our study. This diverges from other studies where RECIST criteria predominantly guided response assessment [6]. This was decided by the investigators of this study because only RECIST criteria might not capture the complete treatment response, particularly in conditions where patient-reported outcomes and symptomatic benefit are crucial indicators of efficacy. Our choice of a 1-year duration for the primary endpoint analysis considered that sorafenib treatment typically spans over a year or longer in DTF. We thus aimed for a comprehensive assessment of treatment response and disease progression within a clinically meaningful timeframe [18]. This duration aligns well with the natural history of DTF and enables us to capture key outcomes related to treatment durability following sorafenib discontinuation.

QoL in patients with DTF on sorafenib is typically lower compared to healthy individuals, as demonstrated in a study conducted at our center [13]. In this study, many burdensome symptoms associated with sorafenib improved upon discontinuation of the drug. Insomnia, diarrhea and financial difficulties showed significant improvement according to patient reports and there was a significant improvement in physical and social functioning. Also notable was a positive trend in global health status even though statistical significance was not reached. An absolute ten-point difference was noted in individual parameters such as fatigue, insomnia, anorexia, diarrhea and financial difficulties which is a clinically significant change as previously reported [20]. While we utilised the EORTC QLQ-C30 tool, we acknowledge the recently validated GODDESS tool which offers a holistic approach to evaluating treatment response specific to DTF [21]. This questionnaire better aligns with the growing emphasis on patient-centered care and the importance of capturing outcomes that resonate with individuals’ lived experiences.

The study was amended to evaluate the trend in neurocognitive functions by utilising the ACE III questionnaire. No change was observed in the mean score of each parameter amongst the small subgroup of six patients who completed the 1-year follow-up of this component of the study. This contrasts with the observations of Mulder *et al* [7] that learning, memory and executive function can be compromised even at 6 months of treatment with VEGF inhibitors. The ACE III questionnaire will continue to be administered to the cohort until the study is completed.

Despite achieving initial disease stabilisation or partial response with sorafenib, the risk of losing this response upon discontinuation is a pertinent concern in DTF management [19]. Several factors contribute to this possibility, including the unpredictable biological behaviour of DTF, individual patient variability and potential development of treatment resistance. It is encouraging that all patients who resumed sorafenib in our study due to recurrent pain achieved symptom relief and radiological stability.

Our focus on sorafenib discontinuation in DTF highlights the critical need for similar investigations in other therapeutic agents such as nirogacestat. We also recognise the emerging role of T2-weighted MRI volumetry in the assessment of DTF [22]. In our forthcoming future projects on DTF, we plan to incorporate T2 MRI volumetry assessments to evaluate outcomes.

The limitations of our study include a short follow-up period, sole focus on extremity DTF and having less than 50% of the planned sample size which has undergone complete follow-up thus far. However, the strength of this study lies in its novel investigation into the optimal duration of sorafenib treatment for stable extremity DTF, utilising a comprehensive assessment approach including MRI, QoL and cognitive evaluations. By demonstrating that a significant proportion of patients remained progression-free and experienced QoL improvements after discontinuation, our findings suggest that clinicians may consider discontinuing sorafenib after a minimum of 1 year of treatment for extremity DTF if the patient is symptom free.

## Conclusion

Early results from the SORASTOP study demonstrate promising results with stopping sorafenib after at least 1 year of therapy in patients with stable extremity DTF. Though we present results of 20 patients, the disease progression in only 1 patient gives important insights into treatment duration for DTF management. We continue the recruitment of patients to reach the planned sample size and are continuing the follow-up of the study cohort.

## List of abbreviations

ACE III, Addenbrooke’s cognitive examination III; DTF, Desmoid-type fibromatosis; EORTC QLQ-C30, European Organisation for Research and Treatment of Cancer Quality of Life Questionnaire-Core 30; ESAS, Edmonton Symptom Assessment Scale; FAP, Familial adenomatous polyposis; MRI, Magnetic resonance imaging; NSAIDs, Nonsteroidal anti-inflammatory drugs; PFR, Progression-free survival rate; QoL, Quality of life; RECIST, Response evaluation criteria in solid tumours; RFS, Recurrence-free survival.

## Conflicts of interest

The authors declare that they have no competing interests.

## Funding

This research did not receive any specific grant from funding agencies in the public, commercial or not-for-profit sectors.

## Ethics approval and consent to participate

Ethical clearance was taken from the Institute’s ethics committee (IECPG-430/28.07.2021).

## Availability of data and materials

Data from the study population can be made available after a reasonable request to the corresponding author.

## Author contributions

BBG: conceptualisation, study design, data curation, data analysis, investigation, methodology, quality control, writing, original draft; GT: conceptualisation, study design, methodology, manuscript preparation, writing, review and editing; SR: conceptualisation, study design, methodology, data analysis, project administration, resources, supervision, validation, quality control, manuscript preparation, manuscript editing; SK, VK: supervision, validation, visualisation; ED, SG, AB, SB: methodology, project administration; MAK: statistical analysis.

## Figures and Tables

**Table 1.** The demographic details of patients, the tumour site distribution, the state of disease at the time of starting sorafenib and the response to the sorafenib treatment of the patients included in stages 1 and 2 of the SORASTOP study.

Variable	Stage-1 (*N* = 16)	Complete (*N* = 33)
Age in years - median (IQR)	31.5 (23.5–38)	29.5 (23–38)
Gender - *n* (%) Male Female	4 (25%) 12 (75%)	15 (45.5%) 18 (54.5%)
Site of tumor Upper limb Shoulder girdle Superficial trunk Lower limb Pelvic girdle	3 (18.75%) 4 (25.0%) 2 (12.5%) 6 (37.5%) 1 (6.25%)	7 (21.2%) 9 (27.2%) 4 (12.1%) 12 (36.4%) 1 (3.0%)
Tumor state when sorafenib started- *n* (%) Primary**^1^** Recurrent**^2^** Refractory**^3^**	7 (43.75%) 3 (18.75%) 6 (37.5%)	13 (39.4%) 9 (27.3%) 11 (33.3%)
The duration of sorafenib received in months Median (IQR)	-	24 (15.5–33.5)
Response to treatment – *n* (%) CR PR SD	*N* = 16 0 7 (43.75%) 9 (56.25%)	*N* = 33 1 (3.0%) 10 (30.3%) 21 (63.6%)

1: Primary disease – Cases diagnosed initially, where patients began sorafenib as the first line of therapy; 2: Recurrent disease – Cases previously treated with surgical resection but recurred, necessitating the initiation of sorafenib. 3: Refractory disease – Cases where patients were on one or more lines of medical management, with or without prior surgeries and remained symptomatic or showed progression at the time sorafenib treatment was considered

Abbreviations: IQR: Interquartile range, CR: Complete response, PR: Partial response, SD: Stable disease

**Table 2.** The MRI measurement and pain score assessed by the ESAS pain scale.

Parameter	Stage 1 (*N* = 16)	Final (*N* = 33)
MRI measurements at the end of 1 year ≥10% increase in the size of the tumor by 1 year ≥20% increase in the size of the tumor	Evaluable patients = 13 1 (7.69%) 0 (0%)	Evaluable patients = 20 3 (15%) 1 (5%)
The number of patients who remained symptom-free at the end of 1 year	Evaluable patients = 15 6 (40%)	Evaluable patients = 20 8 (40%)
Number of patients who had ≥ 5-point increase in Edmonton pain scale at 1-year	Evaluable patients = 15 2 (19%)*	Evaluable patients = 20 4 (20%)**
Number of patients who had disease progression as defined in the primary objective	Total patients = 16 0 (0%)	Total patients = 20 1 (5%)

Abbreviations: MRI, magnetic resonance imaging; ESAS, Edmonton Symptom Assessment Scale

* None had a ≥10% increase in the size of the tumour

** 1 of these 4 patients had a ≥10% increase in the size of the tumour

**Table 3.** Difference in EORTC QLQ C30 HRQoL parameters (symptom and function scales) of the study population at the 6th and 12th month of stopping sorafenib compared to the value at the time of inclusion in the study.

Parameters	Value at baseline	Value at 6th month	Change from baseline, *p*-value	Value at 12th month	Change from baseline, *p*-value
Symptom scale °Fatigue °Pain °Nausea, vomiting °Dyspnea °Insomnia °Appetite loss °Constipation °Diarrhea °Financial difficulties	27.6 ± 21.0 15.6 ± 22.7 6.9 ± 11.9 8.6 ± 22.7 23.6 ± 33.5 18.3 ± 26.9 6.4 ± 15.9 15.0 ± 26.9 29.0 ± 35.2	17.2 ± 22.7 22.8 ± 24.5 1.6 ± 5.0 4.3 ± 14.2 11.8 ± 22.0 8.6 ± 21.0 4.3 ± 11.3 1.1 ± 5.9 17.2 ± 24.1	0.024 0.108 0.010 0.255 0.019 0.083 0.536 0.007 0.032	15.0 ± 21.1 14.7 ± 20.3 3.7 ± 12.2 1.9 ± 8.0 3.9 ± 11.0 5.5 ± 12.7 1.9 ± 8.0 0 11.7 ± 16.4	0.153 0.864 0.430 0.543 0.011 0.083 0.431 0.024 0.016
Function scale °Physical °Role °Emotional °Cognitive °Social	80.6 ± 16.3 87.6 ± 25.0 74.1 ± 21.6 76.3 ± 20.0 88.2 ± 22.0	88.6 ± 11.6 91.4 ± 16.0 81.2 ± 22.4 86.5 ± 17.4 91.9 ± 19.6	< 0.001 0.214 0.021 0.003 0.147	91.5 ± 9.6 92.6 ± 11.7 84.8 ± 18.3 85.3 ± 17.5 93.1 ± 11.8	< 0.001 0.091 0.154 0.109 0.049

Abbreviation: EORTC QLQ-C30: European Organisation for Research and Treatment of Cancer Quality of Life Questionnaire-Core 30

**Table 4.** Difference in EORTC QLQ C30 HRQoL parameters (Global health status scale) of the study population at the 3rd, 6th and 12th month of stopping sorafenib compared to the value at the time of inclusion in the study.

Global health status	At the time of stopping the sorafenib	At the different fixed time points	*p* value
Baseline versus 3rd month *N* = 26	77.24 ± 21.3	77.23 ± 19.8	1.0
Baseline versus 6th month *N* = 20	78.33 ± 22.8	74.16 ± 24.01	0.309
Baseline versus 9th month *N* = 13	82.69 ± 21.9	76.91 ± 20.45	0.279
Baseline versus 12th month *N* = 13	82.69 ± 21.9	79.48 ± 20.58	0.529

Abbreviation: EORTC QLQ-C30: European Organisation for Research and Treatment of Cancer Quality of Life Questionnaire-Core 30

**Table 5.** Mean score of individual parameters of neurocognitive functions as assessed using the ACE III questionnaire at different points in time and its comparison with the baseline value (At the time of inclusion in the study).

Parameter	At 0th month	At 6th month	*p* value (6th versus 0th month)	At 12th month	*p* value (12th versus 0th month)
Attention	17.1 ± 1.2	16.6 ± 1.4	0.089	16.67 ± 1.2	0.611
Memory	22.1 ± 4.8	23.2 ± 2.7	0.705	23.7 ± 1.7	0.205
Fluency	11.9 ± 2.2	11.0 ± 2.2	0.027	11.5 ± 2.6	0.111
Language	25.4 ± 0.91	25.8 ± 0.414	0.138	25.8 ± 0.408	0.363
Visuospatial abilities	13.3 ± 2.3	13.9 ± 2.6	0.423	14.2 ± 1.5	0.235

Abbreviation: ACE III: Addenbrooke’s neurocognitive examination
